# Stereotactic Body Radiotherapy for Hepatocellular Carcinoma: A Brief Overview

**DOI:** 10.3390/curroncol30020190

**Published:** 2023-02-18

**Authors:** Yukinori Matsuo

**Affiliations:** Department of Radiation Oncology and Image-Applied Therapy, Graduate School of Medicine, Kyoto University, Kyoto 606-8507, Japan; ymatsuo@kuhp.kyoto-u.ac.jp; Tel.: +81-75-751-3762

**Keywords:** liver cancer, hepatocellular carcinoma, stereotactic body radiotherapy, post-treatment images

## Abstract

Stereotactic body radiotherapy (SBRT), a type of external beam radiotherapy, yields local control of hepatocellular carcinoma (HCC) at rates as high as 90%. SBRT has been recognized as an alternative therapy for patients for whom standard modalities such as surgery (resection or transplantation) or ablation are deemed unsuitable. SBRT has the potential to improve the prognosis of HCC, as it can be used as an adjunct to other treatment modalities. The assessment of post-SBRT images of the treated tumor and surrounding normal liver tissue requires special attention. Future research is warranted to determine how best to use SBRT versus other therapies and how to combine them.

## 1. Introduction

Curative treatment for liver cancer is an onerous task. Liver cancer was the third-highest cause of cancer mortality worldwide in 2020, while its incidence is the seventh-highest among various cancers [1]. In Japan, liver cancer is the fifth-leading cause of cancer mortality [2]. The 5-year relative survival approximates 50% for localized cases, but it is less than 20% for advanced cases. Hepatocellular carcinoma (HCC) is the dominant histological subtype (nearly 90%) of liver cancer [3]. Chronic alcohol consumption, non-alcoholic steatohepatitis, and infection by hepatitis B or C virus (HBV or HCV) are the major risk factors of HCC. HBV infection is most dominant in HCC development in Asia and Africa, while chronic HCV infection is the most common in patients with HCC in North America, Europe, and Japan [4].

Several guidelines have been formulated to guide treatment of HCC. The Barcelona Clinic Liver Cancer (BCLC) recently updated its strategy paper for the prognosis and treatment of HCC [5]. The BCLC recommends ablation, surgical resection, and transplantation for very-early to early disease, and transplantation, transarterial chemoembolization (TACE) and systemic treatment for intermediate disease. Currently, radiotherapy is not included in the standard treatment strategy for HCC. The situation is the same in the Japanese Guidelines, which do not allude to any role of radiotherapy in the management of HCC [6]. However, the role of radiotherapy in HCC treatment is being recognized. The American Society for Radiation Oncology (ASTRO) has recently compiled the guideline for external beam radiotherapy (EBRT) for primary liver cancers [7]. The ASTRO guideline refers to the role of EBRT in the treatment of HCC and intrahepatic cholangiocarcinoma, preferred EBRT techniques, regimens, and dose constraints for organs at risk.

Stereotactic body radiotherapy (SBRT) is a type of external beam radiotherapy, which is characterized by the use of larger fractional doses of radiation in fewer fractions compared to conventional radiotherapy, which can yield local control of HCC lesions as high as 90%. The success of SBRT hinges upon an extremely precise delivery of radiation to the lesion. SBRT has been recognized as an alternative therapy for patients in whom standard treatments, such as surgery or ablation, are contraindicated. Retrospective studies have suggested the safety and efficacy of SBRT for the treatment of HCC [8,9,10], which is supported by the results of several prospective studies. In this study, we performed a brief overview of the current status of SBRT for HCC.

## 2. Outcomes after Definitive SBRT for HCC

Selected prospective studies that investigated definitive SBRT for HCC have been summarized in Table 1 and Table 2 [11,12,13,14,15,16,17,18,19,20]. Most participants had a history of intensive treatment for HCC, except those enrolled in the studies by Kimura et al. [11] and Durand-Labrunie et al. [13].

Most studies reported an attainment of local control in 90–95% of cases (Table 2). The overall survival varied among the studies, which seemed to depend on the BCLC stage or baseline liver function. Kimura et al. enrolled 36 patients in their prospective STRSPH trial to evaluate the efficacy and safety of SBRT for previously untreated solitary primary HCC [11]. With the prescribed dose of 40 Gy in five fractions, local control and overall survival at 3 years were 90% and 78%, respectively. Grade 3 or greater toxicities related to SBRT were observed in four patients (11%), including duodenal ulcer, dyspnea/hypoxia, ascites, liver failure, and portal vein thrombosis. The Child–Pugh score was worsened by two points or more in 12 patients (34%). Durand–Labrunie et al. conducted a prospective phase trial of SBRT for newly diagnosed single HCC [13]. Local control was 98%, and overall survival was 72% at 18 months. Most Grade 3 or greater toxicities were abnormalities of liver function tests. Grade 3 ascites and gastrointestinal hemorrhage were observed in one (2%) and one (2%) patient, respectively. Child–Pugh score increases of ≥ 2 points were observed in 5 out of 22 patients (23%) at 18 months.

Three systematic reviews are available on the outcomes (local control, survival, and toxicities) of SBRT for HCC [21,22,23]. The most recent study by Shanker et al., which reviewed publications between January 2005 and December 2019, pooled 2846 patients with 3088 lesions receiving SBRT for HCC from 49 cohorts [21]. The pooled 3-year local control and overall survival were 84.2% (95% confidence interval (CI), 77.9–88.9) and 48.3% (95% CI, 39.0–57.0), respectively. The incidence of grade 3 toxicity was 6.5% in the population-weighted median. That study also evaluated the relationship between the prescribed doses and local control, survival, and toxicity. Local control increased by 0.24% and 0.36% per Gy at 1 and 3 years, while overall survival was prolonged by 0.07% and 0.39%, respectively, within the range of 40–83.3 Gy with an equivalent dose in 2-Gy fractions, which corresponds to 30–50 Gy in five fractions. The frequency of grade 3 toxicity also increased by 0.03% per Gy, which was relatively smaller than the effects on local control or survival.

A dose-response relationship for local control and survival has been demonstrated in SBRT for HCC. Jang et al. evaluated 108 patients with HCC who received SBRT with a prescribed dose ranging from 33 to 60 Gy in three fractions [24]. Local control and survival were significantly different between doses of >54 Gy, 45–54 Gy, and <45 Gy. However, a trade-off for high-dose SBRT is a risk of severe toxicity, as discussed below.

## 3. Toxicities after SBRT for HCC

The most common toxicity after liver SBRT was deterioration in liver function, followed by gastrointestinal (GI) toxicity. The incidence of severe toxicities ranged from 3% to 30% (Table 2). The difference in the incidence may be attributed to the variation in the baseline liver function, tumor size, and the definition of treatment-related toxicity.

The definition of liver toxicity has evolved from “classic” radiation-induced liver disease (RILD) to “non-classic” RILD. Classic RILD traditionally refers to toxicities, including anicteric hepatomegaly, ascites, and elevated liver enzymes, due to whole-liver irradiation. It has become a rare event in the era of SBRT. Non-classic RILD is characterized by markedly elevated serum transaminases and jaundice. The most common criteria is an increase in the Child–Pugh score of two points or more [25]. The baseline liver function is one of the most important factors for liver toxicity after SBRT. Patients with baseline Child–Pugh class B8 or above are reported to be at a very high risk of toxicity [26,27]. From the results of their prospective phase I study, Cárdenes et al. found a Child–Pugh score of 8 or greater as a risk factor for grade 3 or worse liver toxicity or death within 6 months after SBRT for HCC [26]. The other factor is the dose to the uninvolved liver. The ASTRO Clinical Practice Guideline proposed different dose constraints among non-cirrhosis, Child–Pugh class A, and class B7 [7]. For a mean dose to the whole liver in a 5-fraction schedule, the proposed constraints are 15–18 Gy, 13–15 Gy, and 8–10 Gy, respectively. The guideline recommends the dose to the coolest subvolume of the liver (DCx) [28] to be limited to DC700cc < 21 Gy for non-cirrhosis, DC700cc < 15 Gy for Child–Pugh class A, and DC500cc < 10 Gy for Child–Pugh class B7, respectively.

GI toxicity manifests as ulcer, fistula, or bleeding. The incidence of grade 3 GI toxicity was reported to be 5–10% after SBRT [29]. The proximity of a tumor to the GI structures should be taken into account in planning SBRT for HCC. Several dose constraints are proposed to reduce the risk of such severe GI toxicities [7]. For example, the dose to the hottest 0.03 cc subvolume of the stomach and duodenum should be less than 32 Gy, according to the ASTRO guideline.

## 4. Comparison or Combination with Other Treatment Modalities

Ablation, including radiofrequency ablation (RFA), is recommended for small (<2 cm) HCCs, according to the BCLC strategy [5]. However, the application of RFA is contraindicated for tumors located in proximity to the major vessels or hilum. The application of RFA may be difficult for tumors located near the diaphragm. SBRT is a good alternative to RFA in such situations [30]. So far, no randomized trials are available that directly compare SBRT with RFA. Some studies have compared survival between SBRT and RFA using propensity-score-based techniques [31,32]. Hara et al. evaluated patients with ≤ 3 HCCs with diameters of 3 cm or less who were treated with RFA or SBRT [31]. The comparison of 212 propensity-score-matched patients revealed that overall survival was comparable between the two treatment modalities (69.1% vs. 70.4% at 3 years). Three meta-analyses of studies comparing SBRT with RFA have been published recently [33,34,35]. Local control of HCC by SBRT was reportedly equivalent or superior to RFA. However, survival after SBRT was inferior to that after RFA in two of the three meta-analyses, which may be attributed to the difference in tumor burden or baseline liver function.

TACE is preferred for BCLC-stage B HCC. A few studies have compared SBRT with TACE [36,37]. Sapir et al. evaluated 209 patients with 1–2 tumors who received TACE or SBRT [36]. The inverse probability of treatment weighting based on the propensity score was applied to the patient cohort to limit selection bias between the two treatment groups. SBRT provided a better local control (91% vs. 23% at 2 years). The difference in survival between SBRT and TACE was not significant. SBRT can be an alternative treatment in some patients with indications for TACE.

Systemic therapy is recommended for patients with advanced-stage HCC. The combination of atezolizumab and bevacizumab has been established as a standard regimen for patients with advanced-stage HCC, according to a randomized phase 3 study, which demonstrated a better overall and progression-free survival with this regimen than with sorafenib [38]. The role of SBRT in advanced-stage disease is also being investigated. The results of NRG/RTOG 1112 were disclosed at the 2022 Annual Meeting of the American Society for Radiation Oncology [39]. NRG/RTOG 1112 is a phase 3 trial that compared SBRT followed by sorafenib with sorafenib alone in patients with advanced HCC. The addition of SBRT improved overall survival, progression-free survival, and the time to progression compared to sorafenib alone, without a significant increase in adverse events. The addition of SBRT to immune checkpoint inhibitors is currently under investigation.

SBRT is also used as bridge therapy for patients awaiting liver transplantation for HCC [40,41,42]. Katz et al. retrospectively reviewed 18 patients who underwent stereotactic hypofractionated radiation therapy for HCC as bridge therapy [40]. None of the patients developed severe gastrointestinal toxicity or radiation-induced liver disease. Eleven patients underwent liver transplantation at a median of 6.3 months after the completion of radiotherapy. All patients were alive after liver transplantation or hepatic resection at a median follow-up of 19.6 months.

## 5. Imaging after SBRT for the Liver

The assessment of post-SBRT images warrants attention to changes in the treated tumor tissue [43,44,45] and surrounding normal liver [46,47,48,49].

A size-based evaluation of the tumor response, such as the modified response evaluation criteria in solid tumors (mRECIST [50]) and the response evaluation criteria in cancer of the liver (RECICL [51]), is not suitable to SBRT. During the first 6 to 12 months, the post-SBRT evaluation should be based on nonenhancement of the tumor [43]. Residual early enhancement disappears within 6 months in most cases [44]. Although the response rate of hypervascular HCC after SBRT increases for 2 years, enhancement persists for more than 2 years in some tumors [45].

**Figure 1:**
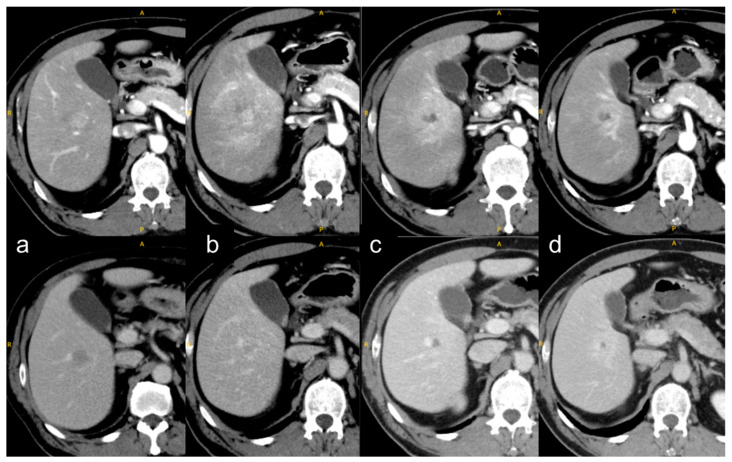
**Focal liver reaction and its time course.** (**a**) Before SBRT, a tumor showed early-phase enhancement (top row) and portal-phase washout (bottom row); (**b**) at 1 month after SBRT, a focal liver reaction with early-phase enhancement around the tumor, corresponding to a 20-Gy irradiated area (not shown here); (**c**) at 4 months, the focal liver reaction shrank and the early-phase enhancement of the tumor disappeared; and (**d**) at 8 months, the focal liver reaction shrank further. The yellow letters A, P, and R indicate anterior, posterior, and right, respectively.

SBRT induces a phenomenon called the “focal liver reaction,” which entails a focal radiation reaction to the surrounding normal liver (Figure 1). The focal liver reaction appears within a 30-Gy irradiated area at a median of 6 months [46]. The reaction is usually classified into three types, but the classification method differs slightly among various studies [47,48,49]. Kimura et al. classified dynamic-computed-tomography appearances into the following three types: type 1, hyperdensity in all enhanced phases; type 2, hypodensity in the arterial and portal phases; and type 3, isodensity in all enhanced phases [49]. The type 2 or 3 appearances were converted into type 1 over time, especially in patients with Child–Pugh class A. Type 3 was associated with Child–Pugh class B.

## 6. Conclusions

With its high local control rate and acceptable toxicities, SBRT has the potential to improve the prognosis of HCC when used in cases that are difficult to treat with other therapies. Future research is warranted to determine how best to use SBRT versus other therapies and how to combine them.

## Figures and Tables

**Table 1:** Patient characteristics in selected studies of stereotactic body radiotherapy (SBRT) for hepatocellular carcinoma (HCC).

Authors (Year)	Study Type	No. of Patients (Lesions)	Age (Years) *	BCLC Stage	Tumor Size (mm) *	Child–Pugh Class	Prior Tx
Kimura et al. (2021) [11]	Phase II	36 (36)	73.5 (57–85)	0: 12 A: 16 C: 8	23 (10–50)	A: 33 B7: 2 B8: 1	0%
Yoon et al. (2020) [12]	Phase II	50 (53)	64 (41–74)	NA	13 (7–31)	A: 50	96%
Durand–Labrunie et al. (2020) [13]	Phase II	43 (43)	72 (43–91)	NA	28 (10–60)	A: 37 B7: 3 B8-9: 2	0%
Jang et al. (2020) [14]	Phase II	65 (73)	61 (44–84)	0: 25 A: 32 B: 4, C: 4	24 (10–99)	A: 64 B7: 1	100%
Park et al. (2020) [15]	Phase II	290 (319)	61 (36–90)	NA	17 (7–60)	A: 250 B: 40	97%
Kim et al. (2019) [16]	Phase I/II	32 (35)	59.5 (42–83)	A: 31 C: 1	21 (10–45)	A: 32	16%
Takeda et al. (2016) [17]	Phase II	90 (90)	73 (48–85)	0: 31 A: 45 C: 16	23 (10–40)	A: 82 B7: 7 B8: 1	64%
Lasley et al. (2015) [18]	Phase I/II	59 (65)	61 (24–86)	NA	NA	A: 38 B7: 17 B8+: 4	15%
Bujold et al. (2013) [19]	Phase I/II	102	69 (40–90)	A/B: 35 C: 67	72 (14–231)	A: 102	52%
Kang et al. (2012) [20]	Phase II	47 (55)	NA	A: 8 B: 31 C: 8	29	A: 41 B7: 6	57%

*Abbreviations:* BCLC = Barcelona Clinic Liver Cancer, Tx = treatment, NA = not available. * The values represent the median (range).

**Table 2:** Clinical outcomes of the studies depicted in Table 1.

Authors	SBRT Dose	Median Follow-Up [Months]	Local Control at 2 Years	Overall Survival at 2 Years	Toxicity (Grade 3+)
Kimura et al. [11]	40 Gy/5 fr	20.8	90%	84%	11%
Yoon et al. [12]	45 Gy/3 fr	47.8	100%	96%	4%
Durand–Labrunie et al. [13]	45 Gy/3 fr	48	94%	69%	31%
Jang et al. [14]	45–60 Gy/3 fr	41	97%	84%	3% (1 year)
Park et al. [15]	30–60 Gy/3 fr	38.2	91.3% (5 years)	44.9% (5 years)	2.8%
Kim et al. [16]	36–60 Gy/4 fr	27	25–94%	81.3%	28%
Takeda et al. [17]	35–40 Gy/5 fr	41.7	96.3% (3 years)	66.7% (3 years)	8.9%
Lasley et al. [18]	48 Gy/3 fr (CP-A), 40 Gy/3 fr (CP-B)	33.3 (CP-A), 46.3 (CP-B)	91% (CP-A), 82% (CP-B)	72% (CP-A), 32.7% (CP-B)	11% (CP-A), 38% (CP-B)
Bujold et al. [19]	24–56 Gy/6 fr	31.4	87% (1 year)	MST 17 months	10%
Kang et al. [20]	42–60 Gy/3 fr	17	94.6%	68.7%	15%

*Abbreviations:* fr = fraction, CP = Child–Pugh, MST = median survival time.
